# Supplementary material for: Transplacental transmission of SARS-CoV-2 infection
Source: Nat Commun. 2020 Jul 14;11:3572. doi: 10.1038/s41467-020-17436-6 (PMC7360599; doi:10.1038/s41467-020-17436-6)
Supplement: Supplementary file 2 — Reporting Summary [file 41467_2020_17436_MOESM2_ESM.pdf]

nature research

Corresponding author(s): Daniele De Luca
Last updated by author(s): 2020/04/21

Reporting Summary

Nature Research wishes to improve the reproducibility of the work that we publish. This form provides structure for consistency and transparency in reporting. For further information on Nature Research policies, see Authors & Referees and the Editorial Policy Checklist .

Statistics

For all statistical analyses, confirm that the following items are present in the figure legend, table legend, main text, or Methods section.

n/a	Confirmed	
☒	☐	The exact sample size (*n*) for each experimental group/condition, given as a discrete number and unit of measurement
☒	☐	A statement on whether measurements were taken from distinct samples or whether the same sample was measured repeatedly
☒	☐	The statistical test(s) used AND whether they are one- or two-sided<br>*Only common tests should be described solely by name; describe more complex techniques in the Methods section.*
☒	☐	A description of all covariates tested
☒	☐	A description of any assumptions or corrections, such as tests of normality and adjustment for multiple comparisons
☒	☐	A full description of the statistical parameters including central tendency (e.g. means) or other basic estimates (e.g. regression coefficient) AND variation (e.g. standard deviation) or associated estimates of uncertainty (e.g. confidence intervals)
☒	☐	For null hypothesis testing, the test statistic (e.g. *F*, *t*, *r*) with confidence intervals, effect sizes, degrees of freedom and *P* value noted<br>*Give P values as exact values whenever suitable.*
☒	☐	For Bayesian analysis, information on the choice of priors and Markov chain Monte Carlo settings
☒	☐	For hierarchical and complex designs, identification of the appropriate level for tests and full reporting of outcomes
☒	☐	Estimates of effect sizes (e.g. Cohen's *d*, Pearson's *r*), indicating how they were calculated

Our web collection on statistics for biologists contains articles on many of the points above.

Software and code

Policy information about availability of computer code

Data collection	N/A
Data analysis	N/A

For manuscripts utilizing custom algorithms or software that are central to the research but not yet described in published literature, software must be made available to editors/reviewers. We strongly encourage code deposition in a community repository (e.g. GitHub). See the Nature Research guidelines for submitting code & software for further information.

Data

Policy information about availability of data

All manuscripts must include a data availability statement. This statement should provide the following information, where applicable:

- Accession codes, unique identifiers, or web links for publicly available datasets
- A list of figures that have associated raw data
- A description of any restrictions on data availability

All data generated or analyzed during this study are included in this published article (and its supplementary information files).

Field-specific reporting

Please select the one below that is the best fit for your research. If you are not sure, read the appropriate sections before making your selection.

☒ Life sciences ☐ Behavioural & social sciences ☐ Ecological, evolutionary & environmental sciences

For a reference copy of the document with all sections, see nature.com/documents/nr-reporting-summary-flat.pdf

Life sciences study design

All studies must disclose on these points even when the disclosure is negative.

Sample size	NA
Data exclusions	NA
Replication	NA
Randomization	NA
Blinding	NA

Reporting for specific materials, systems and methods

We require information from authors about some types of materials, experimental systems and methods used in many studies. Here, indicate whether each material, system or method listed is relevant to your study. If you are not sure if a list item applies to your research, read the appropriate section before selecting a response.

Materials & experimental systems

n/a	Involved in the study	
☐	☒	Antibodies
☒	☐	Eukaryotic cell lines
☒	☐	Palaeontology
☒	☐	Animals and other organisms
☐	☒	Human research participants
☒	☐	Clinical data

Methods

n/a	Involved in the study	
☒	☐	ChIP-seq
☒	☐	Flow cytometry
☒	☐	MRI-based neuroimaging

Antibodies

Antibodies used	CD68 (Dako PG-M1, 1:200), CD163 (Leica 10D6, 1:200), CD20 (Dako L26, 1:400), CD3 (Dako F7.2.38, 1:50), CD5 (Novocastra 4C7, 1:50), CMV (Dako CCH2+DDG9, 1:1), Parvo virus (AbcVs, Abc10-P038), SARS-CoV-2 (Abclonal, rabbit pAB, 2019-nCoV N Protein, 1:2400).
Validation	These were commercially available antibodies, commonly used in clinical diagnostics in Pathology Lab. Validation details of each primary antibody are available on the manufacturers' website.

Human research participants

Policy information about studies involving human research participants

Population characteristics	case report of transplacental transmission of SARS-CoV-2 from a pregnant woman affected by COVID-19 to her offspring
Recruitment	simple observation of an unique case during the pandemics
Ethics oversight	Written informed consent was obtained from the woman for the publication of this report. According to French regulation, institutional review board (IRB) approval is not required for case reports, provided that patients' written consent is obtained. The French Ethical Committee for the Research in Obstetrics and Gynecology reviewed the work and confirmed that the IRB approval was unnecessary. The case study was performed in agreement with principles of the Declaration of Helsinki and CARE guidelines

Note that full information on the approval of the study protocol must also be provided in the manuscript.
